# Case report: Type V ophthalmia nodosa induced by pine processionary caterpillar setae with long-term complications

**DOI:** 10.1186/s12348-025-00549-8

**Published:** 2025-11-28

**Authors:** Mingzhe Shi, Shounan Qi, Chenming Wang, Chenguang Wang

**Affiliations:** Department of Ophthalmology, The Second Hospital of Jilin University, Yatai Street, ChangChun, Jilin Province 130000 China

**Keywords:** Ophthalmia nodosa, Caterpillar setae, Pars plana vitrectomy, Cystoid macular edema

## Abstract

**Background:**

Nodular ophthalmia, a rare and specific inflammatory ocular disease, is typically triggered by contact with setae from *Lepidoptera larvae*. Type V according to the Cadera classification represents the rarest and prognostically poorest variant.

**Case report:**

A 55-year-old male developed immediate ocular pain, foreign body sensation, and blurred vision in his left eye following contact with a *Thaumetopoea pityocampa* (pine processionary caterpillar). Despite prompt removal of visible setae and initiation of topical non-steroidal anti-inflammatory drug (NSAID) therapy at a local facility, uncontrolled intraocular inflammation necessitated referral to our institution. Initial ophthalmic examination revealed best-corrected visual acuity (BCVA) of hand motion (HM) at 15 cm in the left eye, conjunctival injection, and significant inflammatory reactions in the anterior chamber, iris, and vitreous. Two setae were identified: one embedded subconjunctivally and another within the anterior chamber near the anterior iris surface. Combined topical and systemic anti-inflammatory/anti-infective therapy, surgical setae extraction, and diagnostic pars plana vitrectomy (PPV) were performed, achieving control of the intraocular inflammation. At 16 months postoperatively, persistent mild anterior chamber inflammation, cystoid macular edema (CME), epiretinal membrane (ERM), and complicated cataract were observed. At the final 48-month follow-up, BCVA in the left eye stabilized at 0.1.

**Conclusion:**

Precise localization of setae-induced nodular ophthalmia requires comprehensive physical examination and appropriate imaging. Severe posterior segment inflammation mandates prompt surgical setae removal to preserve visual potential. Long-term, meticulous postoperative surveillance is crucial to monitor inflammatory sequelae and intervene promptly for complications.

**Clinical trial number:**

Not applicable.

## Introduction

Nodular ophthalmia is a rare and distinct inflammatory ocular condition, most commonly incited by contact with setae from *Lepidoptera larvae* Sridhar and Ramakrishnan [1]. Setae are defensive hairs on the body surface of *Lepidoptera larvae*, characterized by their slender morphology and covered with tiny barbs. These barbed setae can embed within the conjunctiva or cornea, triggering an inflammatory cascade characterized by pain, foreign body sensation, lacrimation, and conjunctival hyperemia Doshi et al. [2]. Subsequent eye rubbing or ocular movements can facilitate setae migration into the anterior chamber or through the sclera into the vitreous cavity Gonzalez-Martin-Moro et al. [3], potentially inciting intraocular inflammation or diverse pathologies including iridocyclitis, iris nodules, vitritis, or chorioretinitis Doshi et al. [2].

This report details an unusual case where a patient, despite undergoing prompt surgical removal of accessible corneal and conjunctival setae following pine processionary caterpillar contact, developed severe Type V nodular ophthalmia within 14 days due to remaining setae. Vitrectomy was required for definitive setae clearance and inflammation control. Remarkably, over 48 months of follow-up after complete setae removal, the patient exhibited persistent, recurrent low-grade anterior chamber and vitreous cavity inflammation, complicated cataract, CME, and ERM. This atypical course underscores that even minute remaining intraocular setae can incite significant inflammation leading to profound and sustained visual impairment.

## Case report

A 55-year-old male forestry worker presented with conjunctival injection, ocular pain, and blurred vision in his left eye immediately after exposure to a pine processionary caterpillar. Initial management at a local hospital involved topical NSAID therapy and surgical removal of one conjunctival seta. However, symptoms worsened with progressive visual decline, prompting referral to our institution 14 days post-exposure.

**Fig. 1:**
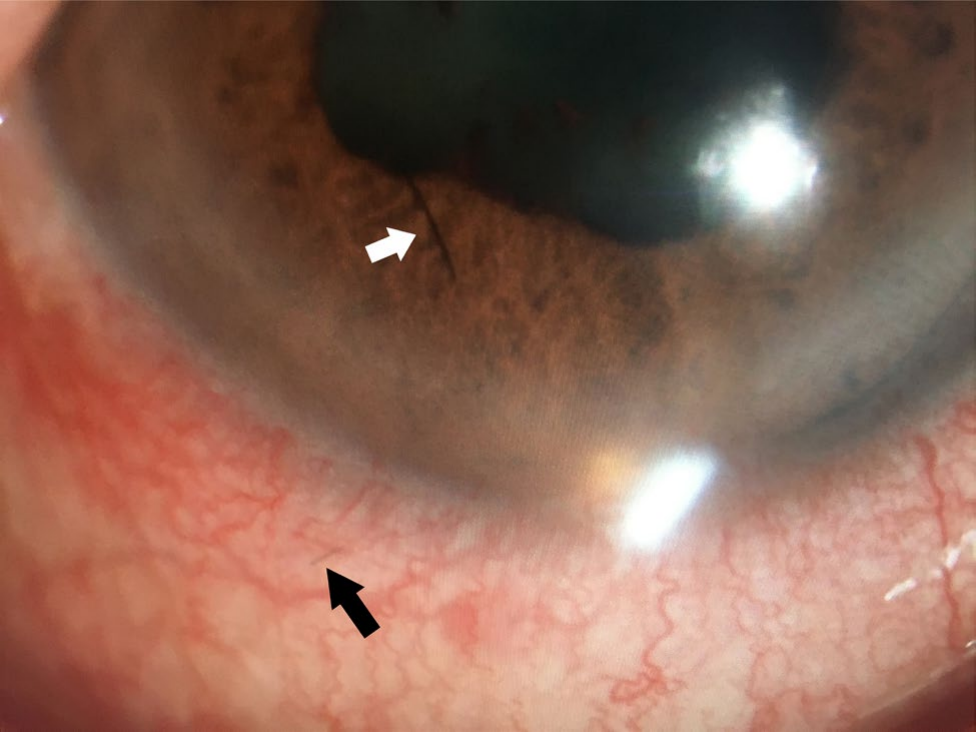
Slit-lamp biomicroscopy of the left eye (16$\times$ magnification) showing a black linear foreign body on the iris surface at the 7 o’clock position (white arrow), and a second subconjunctival black linear foreign body 5 mm from the limbus at the 7 o’clock position (black arrow)

**Fig. 2:**
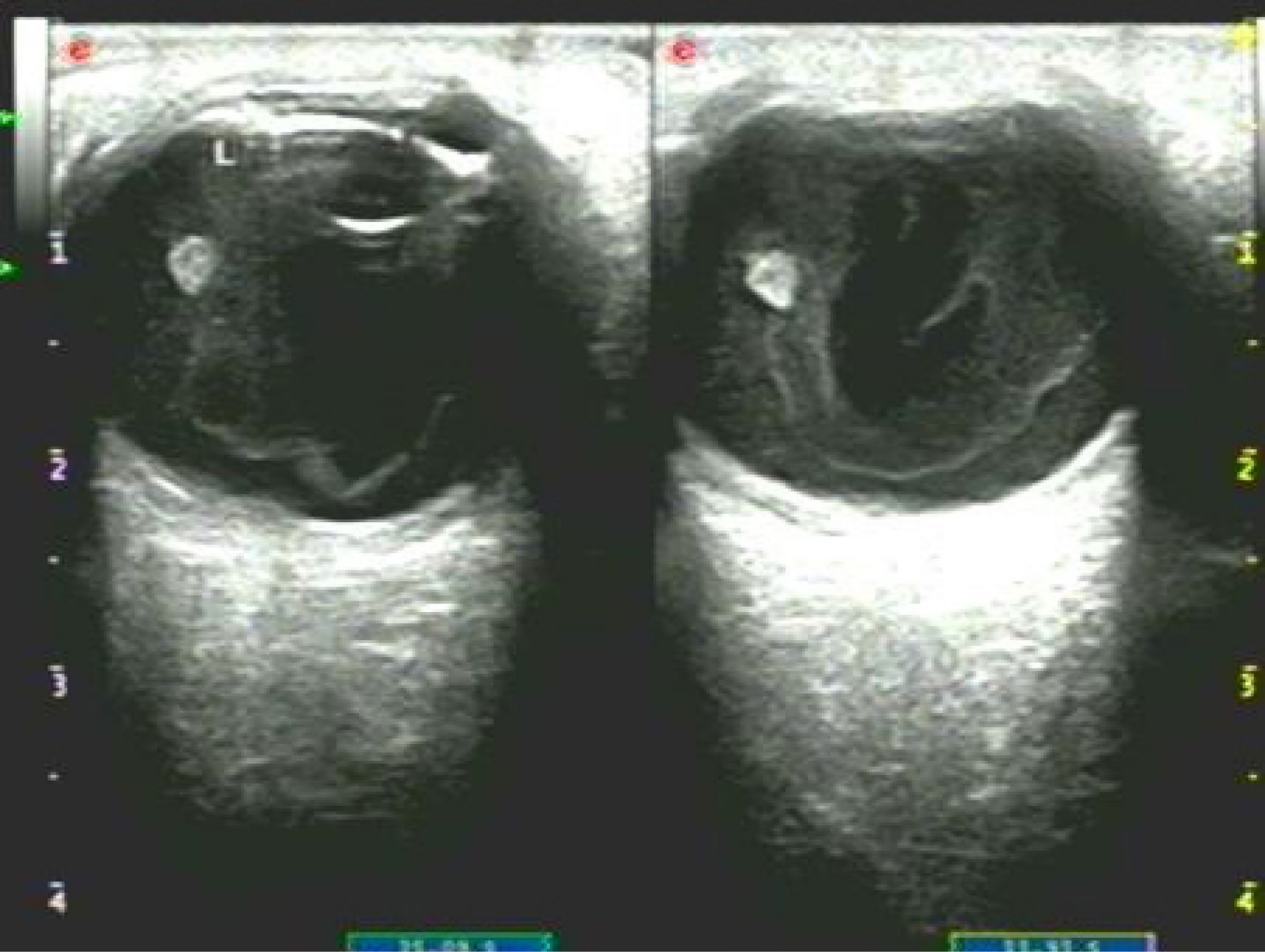
Color Doppler ultrasonography (CDU) of the left eye demonstrating vitreous opacities (medium-to-low reflective echoes)

**Fig. 3:**
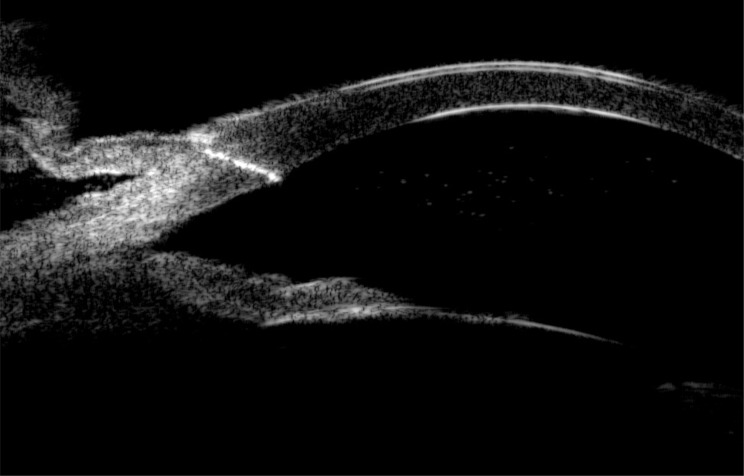
Ultrasound biomicroscopy (UBM) of the left eye (7 o’clock cross-section) revealing a strong linear echo 5 mm from the limbus, consistent with the subconjunctival seta

Comprehensive ophthalmic evaluation upon admission revealed: BCVA - Right Eye (RE): 1.0; Left Eye (LE): HM/15 cm. Intraocular pressure (IOP) - RE: 8 mmHg; LE: 18 mmHg. Slit-lamp biomicroscopy of the LE showed palpebral and bulbar conjunctival injection. A single black, linear foreign body (suspected seta) was located subconjunctivally 5 mm from the limbus at the 7 o’clock position (Fig. 1, black arrow). The anterior chamber exhibited 3+ cells and posterior synechiae inferiorly. A second black, linear foreign body (suspected seta) was visualized within the anterior chamber near the anterior iris surface at 7 o’clock (Fig. 1). Dense vitritis precluded clear fundus view. Ancillary imaging was performed: Color Doppler ultrasonography (CDU) revealed vitreous opacities with medium-to-low reflective echoes (Fig. 2). Ultrasound biomicroscopy (UBM) confirmed the subconjunctival seta’s position and detected a strong linear echo at the 7 o’clock position, 5 mm from the limbus (Fig. 3), ruling out additional occult anterior segment setae.

A diagnosis of nodular ophthalmia was established. Initial medical management included: Topical anti-inflammatory − 1% prednisolone acetate eye drops (QID), 0.1% pranoprofen eye drops (BID); Prophylactic anti-infective − 0.488% levofloxacin eye drops (QID) and intravenous levofloxacin (0.5 g daily); Cycloplegia − 1% atropine sulfate eye gel (BID); and Ocular lubrication − 0.1% sodium hyaluronate eye drops (QID).

**Fig. 4:**
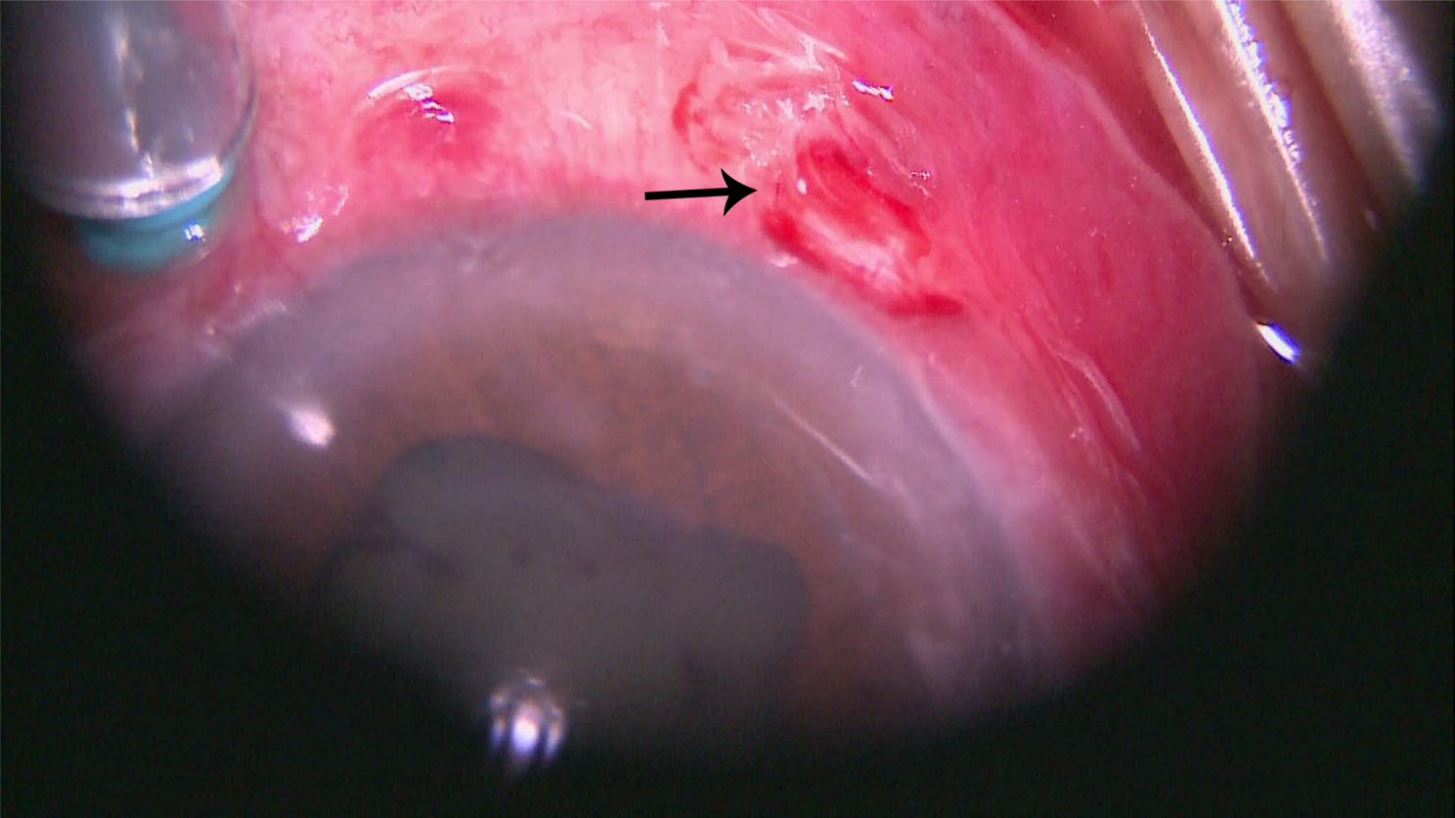
Mirrored intraoperative photograph of the left eye (16$\times$ magnification, surgical microscope) showing removal of the subconjunctival seta using forceps (arrow)

**Fig. 5:**
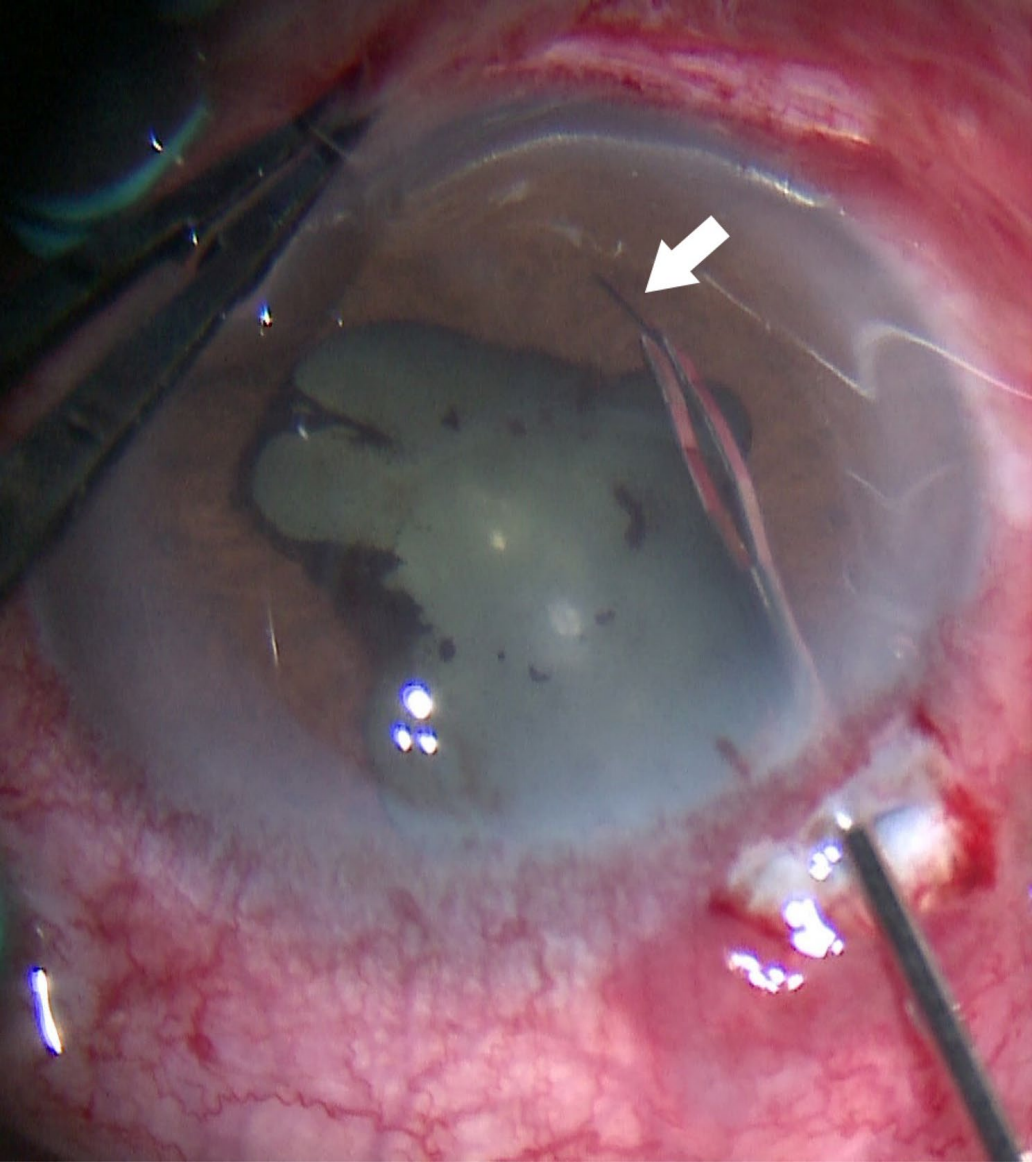
Mirrored intraoperative photograph of the left eye (16$\times$ magnification, surgical microscope) depicting extraction of the intracameral seta via limbal incision using micro-forceps (arrow)

**Fig. 6:**
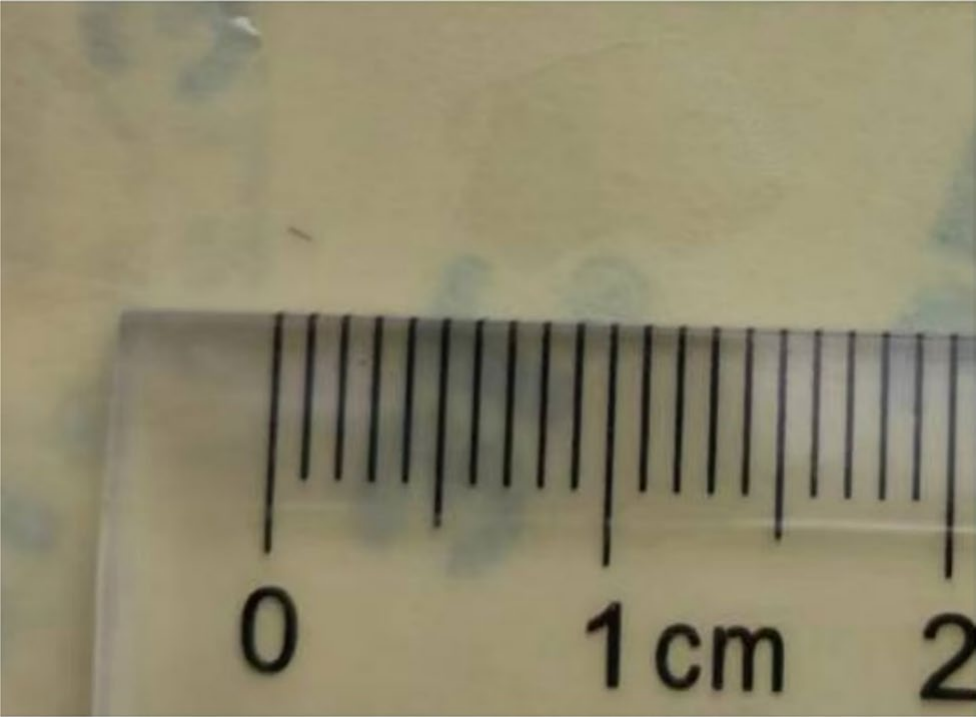
Retrieved intracameral seta from the left eye (approx. 0.6 mm length)

**Fig. 7:**
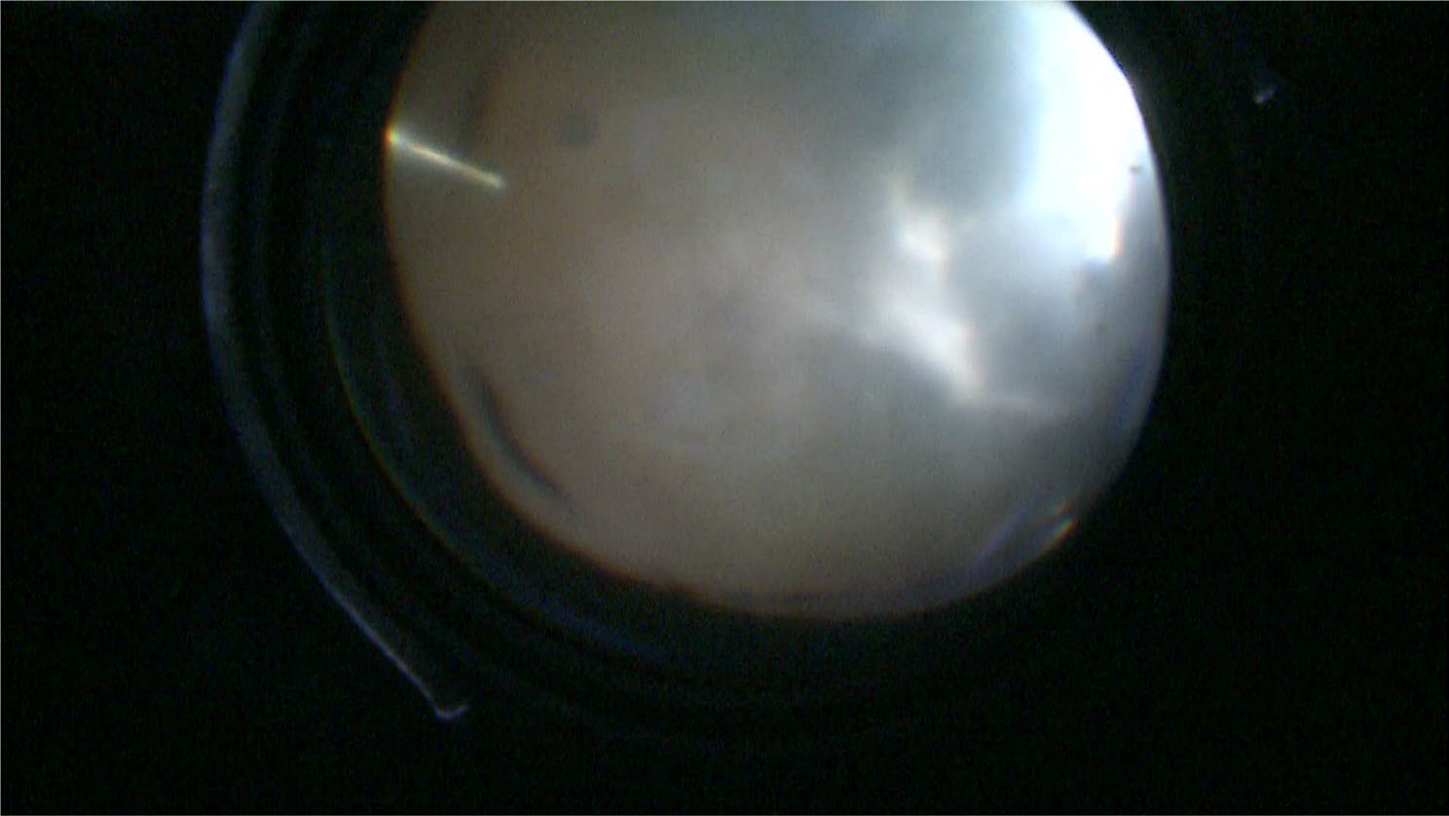
Mirrored intraoperative view of the left eye (surgical microscope with panretinal lens) during pars plana vitrectomy, showing extensive vitreous inflammatory exudates

Definitive management involved combined pars plana vitrectomy (PPV) and intraocular foreign body (IOFB) extraction. Via a limbal conjunctival incision at 7 o’clock, the subconjunctival seta was removed with forceps (Fig. 4). The intracameral seta was extracted through a limbal corneal incision using micro-forceps (Figs. 5 & 6). The lens was preserved. Core vitrectomy was performed, clearing extensive vitreous inflammatory exudates (Fig. 7). The posterior pole and peripheral retina appeared intact.

Postoperatively, topical 0.488% levofloxacin (QID) was continued. Vitreous specimens underwent microbiological (bacterial/fungal cultures) and pathological analysis. Cultures were negative; pathology revealed scant lymphocytic infiltration with no additional setae. Two adjunctive periocular injections of dexamethasone (2 mg) were administered on alternate days to manage postoperative inflammation. And before the microbiological results are available, ibuprofen 300 mg is given orally every day. After the microbiological results are available, it is changed to prednisone tablets 1 mg per kilogram orally every day, the dosage is gradually reduced week by week until the oral administration is stopped 2 months after the operation.

**Fig. 8:**
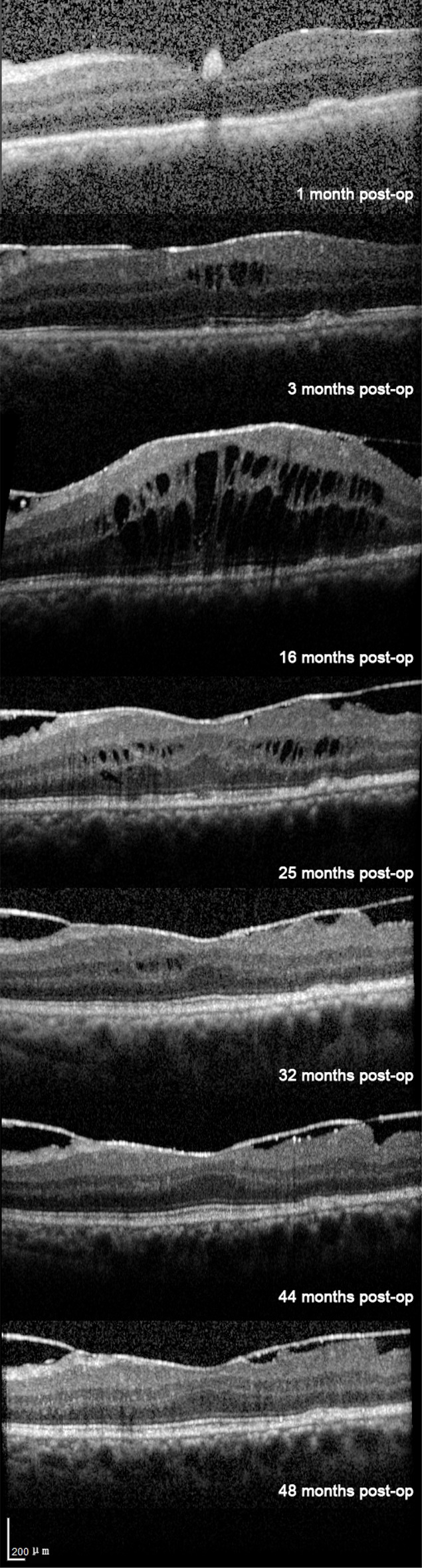
Serial optical coherence tomography (oct) scans of the left eye at the foveal center (identical cross-sectional level, same scale) obtained postoperatively at week 1, months 3, 16, 25, 32, 44, and 48

During the follow-up period, the patient’s left eye BCVA improved to Counting Fingers at 30 cm with an intraocular pressure (IOP) of 8 mmHg by Week 1. Mild inflammation in the anterior chamber and vitreous cavity persisted from 1 month after postoperation, while optical coherence tomography (OCT) showed grossly normal retinal morphology. By Month 3, OCT revealed the development of cystoid macular edema (CME), indicated by intraretinal hyporeflective spaces, and an epiretinal membrane (ERM), with a central retinal thickness (CRT) of 390 $\mu$m (Fig. 8). A significant complicated cataract developed by Month 14, leading to phacoemulsification with intraocular lens implantation, which improved LE BCVA to 0.1, although low-grade inflammation recurred. Conventional anti-infection and anti-inflammatory treatments(1% prednisolone acetate eye drops (QID), 0.488% levofloxacin eye drops (QID)) were given before and after the operation The CME worsened the following month 16, with a peak CRT of 680 $\mu$m (Fig. 8). However, by Month 32, the CME had resolved spontaneously, and the CRT decreased to 250 $\mu$m (Fig. 8). By month 48 (Final Follow-up), BCVA remained stable at 0.1. While CME did not recur, the ERM persisted. Mild, recurrent anterior segment inflammation was still noted.

## Discussion

ON represents a granulomatous inflammatory response of ocular tissues to setae from *Lepidoptera larvae* Sridhar and Ramakrishnan [1]. Upon mechanical stimulation, setae can readily detach from the caterpillar’s integument. Unlike most foreign bodies in the eye, setae exhibit a tendency to migrate inward upon penetrating ocular tissues. This phenomenon occurs partly because the barbs on the setal surface hinder normal expulsion Ascher [4], and partly because inflammatory exudates surrounding the setae may debilitate the tissue, allowing the seta to be propelled by rubbing and physiological ocular and non-ocular pressure changes (respiration, heartbeat, and iris movement)Gundersen et al. [5].

The pathogenic mechanisms primarily involve: (1) Mechanical Injury: Direct physical damage caused by the setae and their barbs. (2) Toxin-Mediated Damage: Toxins (primarily serine proteases) released from the setae hydrolyze extracellular matrix and cell surface proteins, damaging tissues. (3) Allergic Reaction: Specific allergens within setae components (e.g., Tha p1, Tha p2) trigger IgE-mediated type I hypersensitivity; histamine-like substances within setae can directly induce allergy-like symptoms. (4) Inflammatory Reaction: Multiple setal components synergistically activate inflammatory pathways: Setal proteins and toxins directly activate immune cells (lymphocytes, macrophages); serine proteases indirectly amplify inflammation by activating the complement and kinin systems; damaged cells further activate inflammatory cascades; chitin activates macrophages, and its breakdown products promote sustained inflammation Agarwal et al. [6], Berardi et al. [7], Sánchez et al. [8].

The Cadera classification (Types I-V) provides a crucial framework for ON diagnosis and management: Types I-II (conjunctival/corneal involvement and inflammation) constitute the majority of cases; Types III-IV (anterior segment granuloma, iritis) may necessitate surgical setae removal; Type V (vitreous and retinal involvement) is the rarest and carries the poorest prognosis Cadera et al. [9]. The uniqueness of the present case lies in its rapid progression to Type V ON within 14 days despite early removal of ocular surface setae (consistent with initial management for Types I-II), and the subsequent development of recurrent intraocular inflammation persisting for 36 months postoperatively. This underscores the necessity for prolonged surveillance and inflammation control even after apparent foreign body clearance.

The migratory behavior of intraocular setae has been systematically described. Julio et al. proposed a classification based on setal migration pathways, which categorizes the process into initial ocular surface deposition (Phase 1), followed by penetration through the cornea (Phase 2, Route A) or sclera (Phase 2, Route B), and subsequent entry into the anterior (Phase 3, Route A) or posterior segment (Phase 3, Route B) Gonzalez-Martin-Moro et al. [3].

In the present case, surgical intervention retrieved two setae. Their locations prior to removal align with this classification: one seta, preoperatively identified via UBM in the anterior chamber, had migrated through the corneoscleral limbus, corresponding to Route A. The other seta was located in the subconjunctival space without full scleral penetration, consistent with Phase 2, Route B.

This case exhibited severe, rapidly progressive, and protracted inflammation. While previous studies predominantly correlate ON prognosis with the quantity of intraocular setae Sengupta et al. [10], Leclaire et al. [11], and some reports describe setae residing intraocularly for months before causing Type V ON Liu and Jiang [12], Kumar Menia et al. [13], the current case presented uniquely: despite the surgical retrieval of only two setae and the exclusion of infectious endophthalmitis, the patient experienced (1) progression to Type V ON within 14 days, (2) persistent anterior chamber/vitreous cavity inflammation for 16 months postoperatively, (3) CME lasting 32 months (peak CRT 680 $\mu$ m) with ERM, and (4) complicated cataract. We propose several potential explanations for this unusual course: (1) Antigen Persistence: One seta penetrated the limbus; even after removal, remaining setal fragments or antigens embedded within ocular tissues may provide chronic antigenic stimulation. Chitin components could perpetuate chronic inflammation and hypersensitivity. (2) Toxin Persistence: The morphology of the retrieved intracameral seta ( 0.6 mm) aligns with urticating setae. Toxin release explains the intense initial inflammation; residual toxins within ocular tissues post-removal may sustain inflammation, with protease activity contributing to increased vascular permeability (a key factor in CME). (3) Secondary Inflammatory Cascade: Acute-phase inflammation upregulated cytokines and chemokines, inducing lymphocytic infiltration (confirmed on vitreous pathology). This likely activated retinal glial cells (basis for ERM formation) and disrupted lens metabolism (contributing to cataractogenesis).

While prior ON research primarily focuses on acute-phase setae removal Liu and Jiang [12], Zhang et al. [14], Leclaire et al. [11], this case demonstrates that chronic inflammation can persist for many months following the clearance of even minute quantities of toxic setae, leading to irreversible visual impairment (final BCVA 0.1). Limitations of this study include the lack of toxicological analysis of the retrieved setae and the absence of aqueous/vitreous cytokine profiling. Future research should establish standardized toxicological analyses of pine processionary caterpillar setae on ocular tissues and explore targeted anti-inflammatory strategies for both acute and chronic phases of ON.

## Conclusion

The management of this case highlights key lessons: Ocular injury by pine processionary caterpillar setae presents diverse and challenging manifestations. Clinically, setae are highly migratory, often multiple, and frequently occult. Posterior segment involvement is rare but severe. Management mandates: 1. Meticulous History & Examination: Identify the source and potential migration routes. Pay particular attention to the pars plana and anterior chamber angle. 2. Precise Localization: Utilize anterior segment imaging (UBM, AS-OCT) for effective localization of anterior segment setae. While offering lower resolution, color Doppler ultrasonography (CDU) can aid in detecting vitreous setae. 3. Prompt and Complete Removal: Perform definitive setae removal as early as possible after localization. Select the surgical approach (e.g., ocular surface extraction, anterior chamber paracentesis, lens extraction, pars plana vitrectomy) based on setae location. 4. High Index of Suspicion for Intraocular Migration: Even after seemingly complete removal of ocular surface foreign bodies, remain vigilant for intraocular penetration (anterior chamber/posterior segment). Employ thorough imaging (UBM/CDU) to rule out and remove migrated setae. 5. Aggressive Inflammation Management & Long-Term Surveillance: If complete removal is unattainable, implement close follow-up and intensive anti-inflammatory therapy. Monitor for worsening inflammation using posterior segment OCT to detect retinal complications early. Recognize that chronic inflammation can persist long after setae removal, potentially leading to sight-threatening sequelae such as complicated cataract, CME, and ERM. Long-term, meticulous postoperative monitoring is essential to detect and intervene promptly for these chronic complications.

## Data Availability

No datasets were generated or analysed during the current study.
